# Molecular characterization of type 1 porcine reproductive and respiratory syndrome viruses (PRRSV) isolated in the Netherlands from 2014 to 2016

**DOI:** 10.1371/journal.pone.0218481

**Published:** 2019-06-27

**Authors:** J. C. F. M. Dortmans, G. J. Buter, R. Dijkman, M. Houben, T. F. Duinhof

**Affiliations:** GD Animal Health, Deventer, The Netherlands; University of Illinois College of Medicine, UNITED STATES

## Abstract

Porcine reproductive and respiratory syndrome virus (PRRSV) is the causative agent of a devastating pig disease present all over the world. The remarkable genetic variation of PRRSV, makes epidemiological and molecular analysis of circulating viruses highly important to review current diagnostic tools and vaccine efficacy. Monitoring PRRS viruses supports modern herd management by explaining the source of found viruses, either internally or externally from the herd. No epidemiological or molecular study has been published on circulating PRRS-viruses in the Netherlands, since the early nineties. Therefore, the objective of this study is to investigate circulating PRRS-viruses in the Netherlands in 2014, 2015 and 2016 on a molecular level by sequencing ORF2, ORF3, ORF4, ORF5, ORF6 and ORF7. The results demonstrate that the 74 PRRSV strains belong to PRRSV-1, but the diversity among strains is high, based on nucleotide identity, individual ORF length and phylogenetic trees of individual ORFs. Furthermore, the data presented here show that the phylogenetic topology of some viruses is ORF dependent and suggests recombination. The identity of the strain of interest might be misinterpreted and wrong conclusions may be drawn in a diagnostic and epidemiological perspective, when only ORF5 is analyzed, as performed in many routine sequencing procedures.

## Introduction

Porcine reproductive and respiratory syndrome (PRRS) is the most significant swine disease worldwide since its appearance in the eighties, and is endemic in many countries [1], including the Netherlands [2, 3]. The disease is characterized by abortions and weak born piglets, increased mortality in suckling and weaned piglets, and respiratory disease in weaners and finishers [4, 5]. This disease is caused by the PRRS-virus (PRRSV) and this virus also aggravates infections like Influenza A, *Streptococcus suis* and porcine respiratory coronavirus infections [1]. PRRSV belongs to the *Arteriviridae* family within the order *Nidovirales* and contains a single-stranded positive sense RNA genome ranging from 14.9 to 15.5 Kb in length [6, 7]. PRRSV is divided into two genotypes, PRRSV-1, and PRRSV-2 [8]. Both types share only about 50–60% and there is considerable sequence variability within each type [9–12]. Both subtypes now have a worldwide distribution [10, 13–16].

The genome encodes at least 10 open reading frames (ORFs) [17]. ORF1a and ORF1b encode non-structural polyproteins with replicase and polymerase activities. Downstream, ORF2, ORF3 and ORF4 encode for the minor structural glycoproteins GP2, GP3 and GP4, respectively and together these proteins form a trimeric complex that is heavily N-glycosylated and functions in viral entry [17]. In addition to GP2, ORF2 encodes an alternative reading frame (ORF2b) for a small unglycosylated envelope protein (E) [18]. Further downstream, ORF5, ORF6 and ORF7 encode for the major structural proteins GP5, matrix (M) and nucleocapsid (N), respectively [17]. Besides the N-glycosylated GP5, that is involved in cell attachment, another small unglycosylated protein that is required for virus viability is translated from an alternative reading frame of ORF5, designated ORF5a [19].

Monitoring PRRS viruses supports modern herd management by explaining the source of found viruses, either internally or externally from the herd. The remarkable genetic variation of PRRSV [20–22], makes epidemiological and molecular analysis of this virus of high importance to monitor changes of these circulating viruses. Consequently, it is also important to review current diagnostic tools since the variability of PRRSV has impact on the sensitivity and specificity of used ELISAs and PCR tests and may affect vaccine efficacy. Of the Dutch pigs, approximately 95% of the sows and 30% of the piglets is vaccinated with either modified life vaccine (MLV) Porcilis PRRS (MSD), Reprocyc/PRRSFLEX EU (Boehringer Ingelheim), Unistrain (Hipra) or Suvaxyn MLV PRRS (Zoetis). In addition, on a few sow farms the inactivated vaccine Progressis (CEVA) is used.

Since the first description of PRRSV in the Netherlands in 1991 [23] no comprehensive epidemiological or molecular study has been published on circulating PRRS-viruses in the Netherlands except two posters presented at the International Pig Veterinary Society congress in 2008 [24] and 2010 [25]. These studies showed a phylogenetic analysis of ORF5 sequences of Dutch strains from 2004–2009 and concluded that the average sequence similarity with Lelystad virus (<90%) is decreasing over the years. Therefore, the objective of this study is to investigate circulating PRRS-viruses in the Netherlands in 2014, 2015 and 2016 on an extensive molecular level by sequencing ORF2, ORF3, ORF4, ORF5, ORF6 and ORF7. Subsequently, these sequences are compared to PRRSV sequences originating from other European countries and available in GenBank in order to investigate genetic relatedness.

## Material and methods

### Samples

Seventy-four serum samples containing genetic material of PRRS type 1 viruses confirmed by PRRSV genotype specific real-time TaqMan PCR targeting the ORF7 region of the PRRSV genome [26] were selected for further molecular characterization. The samples originated from fifty-four Dutch pig farms, predominantly located in the eastern part of the Netherlands and were collected from and including the years 2014 to 2016.

### Sequencing PRRS viruses

Viral RNA was extracted from serum samples using the MagMAX pathogen DNA/RNA isolation kit as deposited in protocols.io [27] in combination with the semi-automated MagMAX^TM^ Express-96 Deep Well Magnetic Particle Processor (Thermofisher Scientific) according to the manufacturer’s protocol. First-strand cDNA synthesis was done with the SuperScript® III kit (Invitrogen) using the 3’-end poly(dT) reverse transcription (RT)-primer (S1 Table) as described in protocols.io [28, 29]. Subsequently, a long range amplification PCR of ORF2-ORF7 was performed with the AccuPrimeTM Taq DNA Polymerase High Fidelity mix (Invitrogen) using a forward and reverse primer (S1 Table) as deposited in protocols.io [30]. Samples without visible product after PCR were re-tested using a second, and when necessary, a third forward primer. Subsequently, amplicons were sent to BaseClear (Leiden, the Netherlands) for purification and Sanger sequence analysis. PCR amplification primers and Sanger sequence primers are presented in S1 Table.

### Sequence analysis

For each virus, sequences were edited (trimming of the sequence of the primer binding sites) and assembled using Lasergene Seqman Pro version 15 (DNAstar inc. Madison, Wisconsin USA). The 74 ORF2-ORF7 sequences were deposited in GenBank and accession numbers are presented in Table 1. Dutch sequences were aligned with sequences obtained from GenBank and phylogenetic trees were constructed using MEGA6 software by Neighbor-Joining method (1000 replicates for bootstrap). The evolutionary distances were computed by using the Maximum Likelihood method based on the Tamura-Nei model [31]. The trees were drawn to scale, with branch lengths measured in the number of substitutions per site.

**Table 1:** GenBank accession numbers of 74 Dutch PRRSV viruses.

Isolate	GenBank	Isolate	GenBank	Isolate	GenBank	Isolate	GenBank
NL/GD-1-1/2015	MK404230	NL/GD-2-16/2014	MK404249	NL/GD-4-8/2015	MK404268	NL/GD-6-1/2015	MK404287
NL/GD-1-2/2015	MK404231	NL/GD-2-17/2014	MK404250	NL/GD-4-9/2015	MK404269	NL/GD-6-3/2015	MK404288
NL/GD-1-3/2015	MK404232	NL/GD-2-18/2015	MK404251	NL/GD-4-10/2015	MK404270	NL/GD-6-4/2015	MK404289
NL/GD-1-4/2015	MK404233	NL/GD-2-19/2015	MK404252	NL/GD-4-11/2015	MK404271	NL/GD-6-7/2016	MK404290
NL/GD-1-6/2015	MK404234	NL/GD-3-5/2014	MK404253	NL/GD-4-13/2015	MK404272	NL/GD-6-8/2016	MK404291
NL/GD-1-7/2015	MK404235	NL/GD-3-7/2014	MK404254	NL/GD-4-14/2015	MK404273	NL/GD-6-9/2016	MK404292
NL/GD-1-9/2015	MK404236	NL/GD-3-8/2014	MK404255	NL/GD-4-15/2015	MK404274	NL/GD-6-12/2016	MK404293
NL/GD-1-12/2015	MK404237	NL/GD-3-9/2014	MK404256	NL/GD-4-16/2015	MK404275	NL/GD-6-17/2016	MK404294
NL/GD-1-15/2016	MK404238	NL/GD-3-11/2015	MK404257	NL/GD-5-1/2015	MK404276	NL/GD-6-18/2016	MK404295
NL/GD-1-17/2016	MK404239	NL/GD-3-12/2015	MK404258	NL/GD-5-3/2015	MK404277	NL/GD-6-19/2016	MK404296
NL/GD-1-18/2016	MK404240	NL/GD-3-14/2015	MK404259	NL/GD-5-4/2015	MK404278	NL/GD-7-1/2016	MK404297
NL/GD-2-5/2014	MK404241	NL/GD-3-15/2015	MK404260	NL/GD-5-8/2015	MK404279	NL/GD-7-2/2016	MK404298
NL/GD-2-6/2014	MK404242	NL/GD-3-16/2015	MK404261	NL/GD-5-9/2015	MK404280	NL/GD-7-5/2016	MK404299
NL/GD-2-8/2014	MK404243	NL/GD-3-18/2015	MK404262	NL/GD-5-11/2015	MK404281	NL/GD-7-6/2016	MK404300
NL/GD-2-9/2014	MK404244	NL/GD-3-19/2014	MK404263	NL/GD-5-12/2015	MK404282	NL/GD-7-7/2016	MK404301
NL/GD-2-10/2014	MK404245	NL/GD-4-1/2015	MK404264	NL/GD-5-13/2015	MK404283	NL/GD-7-9/2016	MK404302
NL/GD-2-12/2014	MK404246	NL/GD-4-3/2015	MK404265	NL/GD-5-14/2015	MK404284	NL/GD-7-12/2015	MK404303
NL/GD-2-13/2014	MK404247	NL/GD-4-4/2015	MK404266	NL/GD-5-18/2015	MK404285		
NL/GD-2-14/2014	MK404248	NL/GD-4-5/2015	MK404267	NL/GD-5-19/2015	MK404286		

### Genetic variation

The large genetic variation of the Dutch PRRSV strains was studied using a percent identity matrix by determining the pairwise nucleotide identities (including gaps) between different ORFs of different isolates using the online Clustal Omega tool [32].

Because the structure of the viral proteins may affect the infectivity, pathogenicity and viral persistence of the virus [33, 34], individual ORF lengths were determined and putative N-linked glycosylation sites were estimated. Nucleotide sequences were translated using EditSeq version 15 (DNAstar inc. Madison, Wisconsin USA) and putative N-linked glycosylation sites were predicted by means of the NetNGlyc1.0 Server [35]. Furthermore, signal peptides were determined with SignalP 4.0 [36]. The PRRSV-1 strain Lelystad virus (M96262) was used as reference strain to compare similarity scores, ORF lengths and glycosylation sites.

## Results

**Fig 1:**
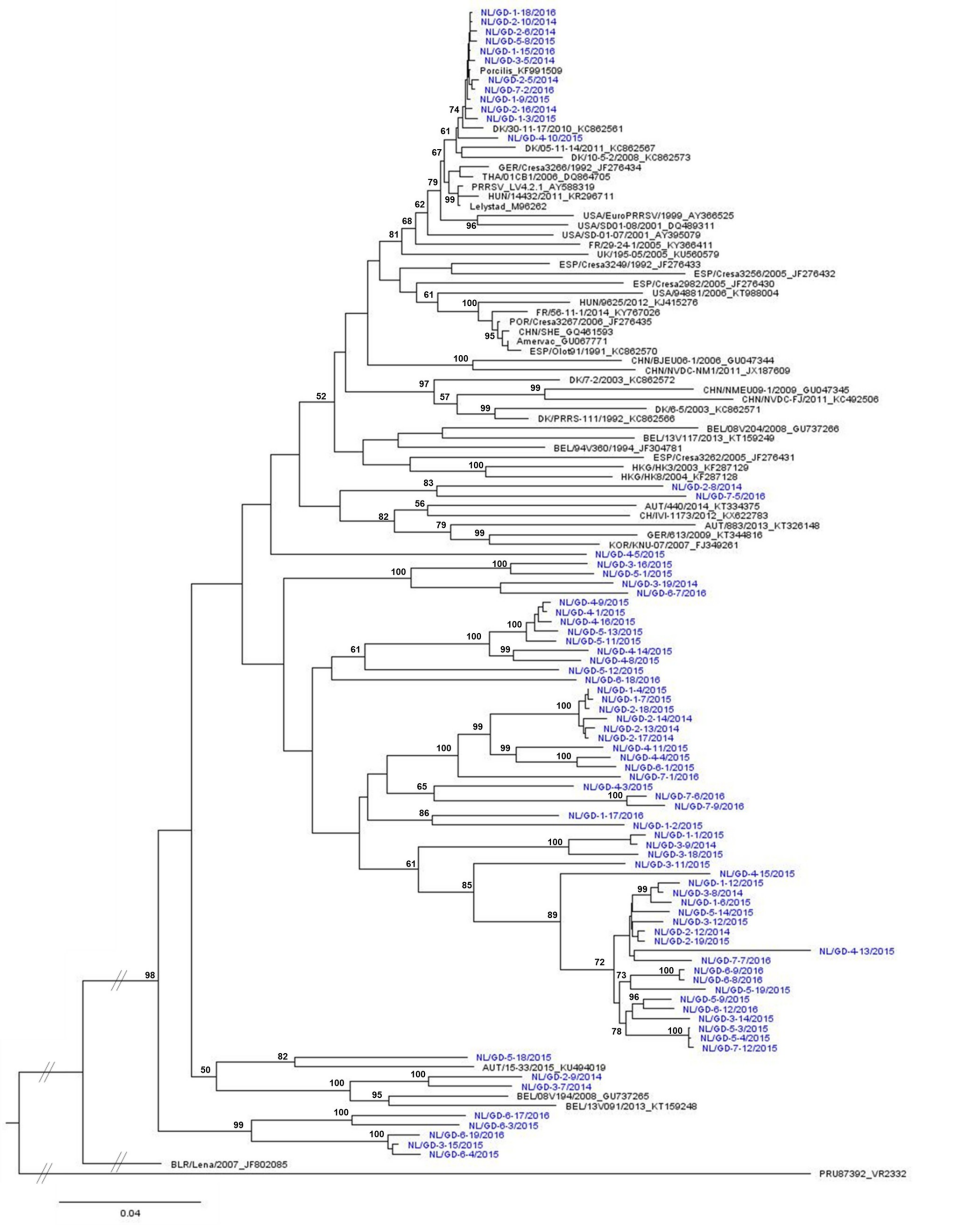
Phylogenetic analysis. Dutch ORF2-ORF7 sequences isolated in 2014–2016 (blue) were compared with representative PRRSV-1 sequences available in GenBank (black). GenBank accession number is given, as well as country/identifier/year, if available. The tree is rooted with type 2 strain VR2332 (PRU87392). The percentage of trees in which the associated taxa clustered together is shown next to the branches (only >50).

To determine the genetic diversity of PRRS viruses in the Netherlands, the ORF2-ORF7 sequences of 74 viruses, originated from different regions were compared with sequences in GenBank. All investigated viruses belong to PRRSV-1 (Fig 1). Twelve viruses had a nucleotide similarity of 98.6–99.8% with Porcilis PRRS vaccine (strain DV; Genbank acc.no. KF991509) and are therefore considered as vaccine derived viruses (similarity >98%). Most other Dutch viruses seem to be divergent from other available sequences in GenBank with only a similarity of 87.5–90.0% with Lelystad virus (GenBank M96262), the type 1 reference strain. As a result, most isolates form a distinct Dutch cluster in the phylogenetic tree, based on the ORF2-ORF7 sequences (Fig 1). Furthermore, within this Dutch cluster a high genetic diversity among the Dutch strains was observed (S2 Table). For instance, nucleotide identity between NL/GD-2-8/2014 (84.7–89.3%), NL/GD-7-5/2016 (84.6–88.3%), NL/GD-4-5/2015 (84.6–89.1%) and NL/GD-6-18/2016 (85.9–89.4%) was below 90% (viruses illustrated in blue, S2 Table). Three viruses were placed with other European strains (Fig 1), however the similarity with these strains is not high: NL/GD-2-9/2014 and NL/GD-3-7/2014 were placed with Belgium strains (GU737265 and KT159248) and are 90–92% similar and NL/GD-5-18/2015 versus the Austrian strain (KU494019) results in a nucleotide identity of only 90%.

The distribution of the frequency of the similarities with Lelystad virus in percentages is presented for the complete ORF2-ORF7 sequence and for the individual ORF genes (S1 appendix). ORF6 and ORF7 have the highest nucleotide identity with Lelystad virus. Although lower similarities can be seen in ORF5, the lowest average similarity with Lelystad virus was observed in ORF3, since five viruses had lower nucleotide identities than 82% (S1 appendix and S2 Table).

**Fig 2:**
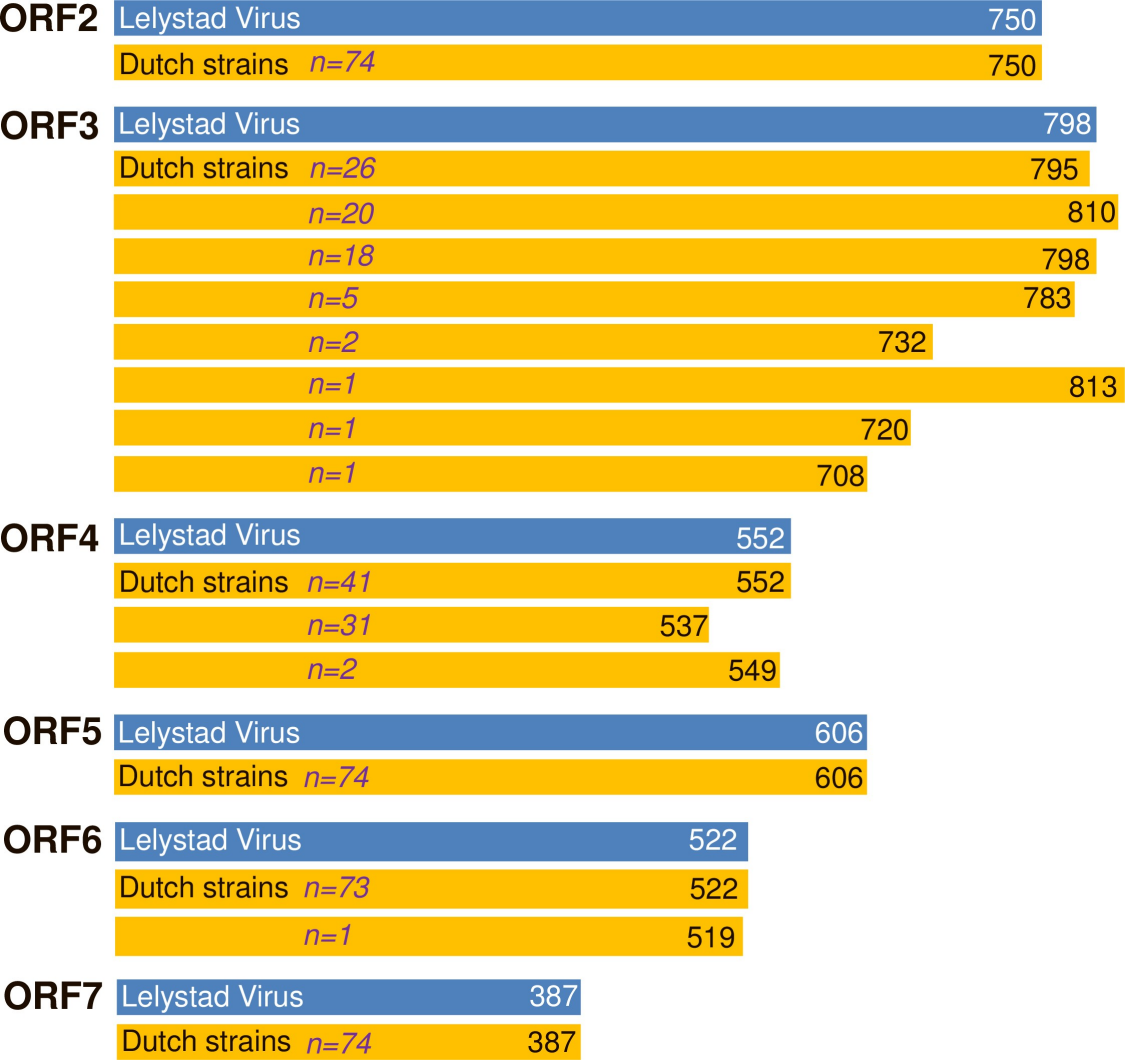
ORF length. Nucleotide length of the open reading frames (ORF) of the 74 isolates (orange) compared to the reference strain Lelystad Virus (GenBank M96262; blue).

While the length of ORF2, ORF5, ORF6 and ORF7 seems conserved compared to the Lelystad reference strain (GenBank: M96262), the length of ORF3 and ORF4 are quite diverse among the isolates (Fig 2 and S3 Table). For ORF3, no less than eight variants in length were observed and five lengths were found more than once (Fig 2). All deletions found in ORF3 are positioned at the 3’-end of this gene and affect automatically ORF4, since the open reading frames overlap. Two viruses (NL/GD-2-8/2014 and NL/GD-3-7/2014) with a short ORF3 length of 732 nucleotides are not caused by deletions, but due to an alternative stop codon. The prediction of the amount of putative N-glycosylation sites for ORF3 varied from 4–7 (S4 Table). For ORF4, three variants in length in the Dutch strains (Fig 2) were observed, and whereas most Dutch viruses contain four N-glycosylation sites, two viruses have 5 putative sites (S4 Table). The one ORF6 sequence (519 nucleotides) with a codon missing (Fig 2), seemed to be unique among the known ORF6 sequences in GenBank.

**Fig 3:**
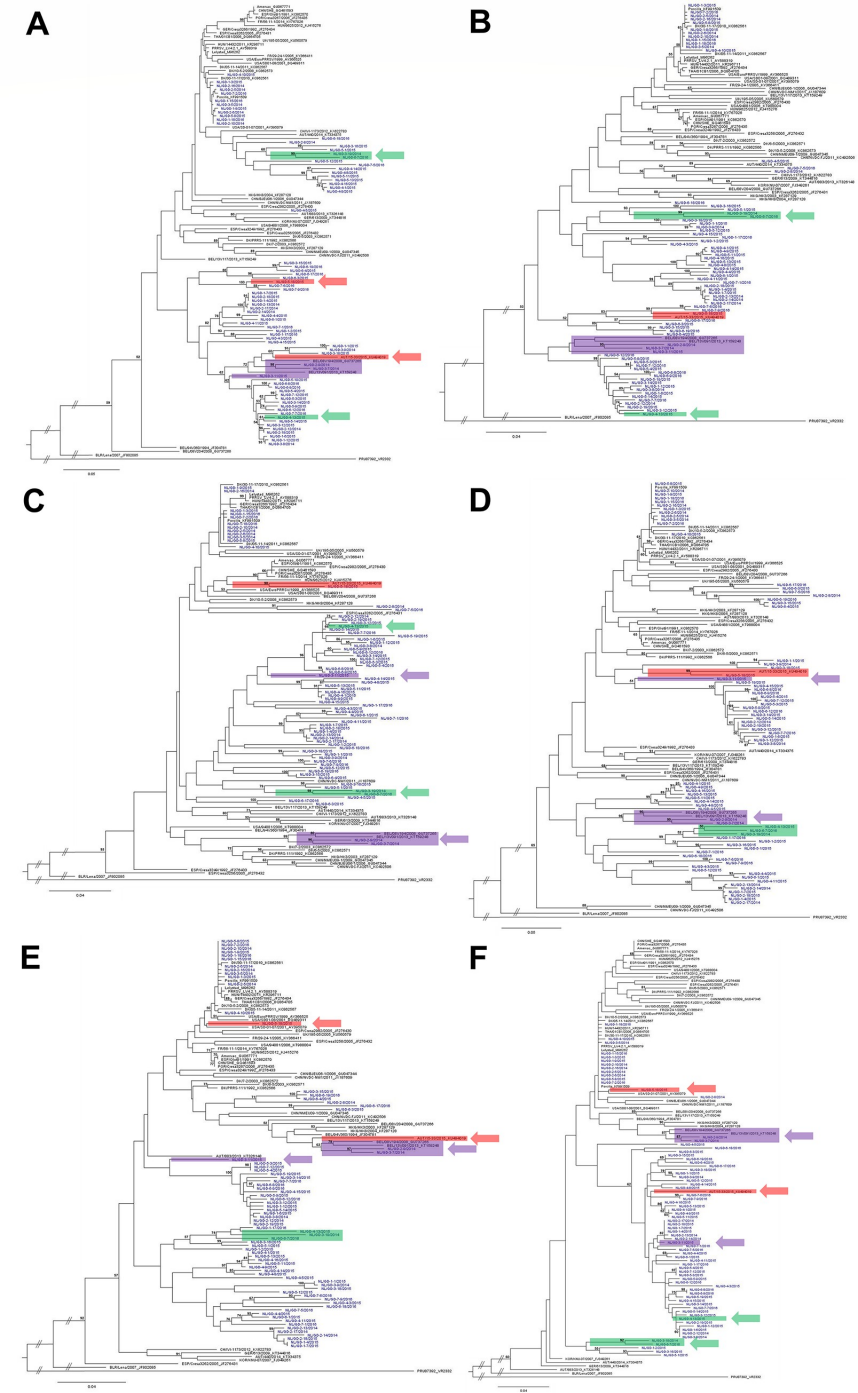
Phylogenetic analysis of individual ORFs. Phylogenetic analysis of Dutch sequences isolated in 2014–2016 (blue) compared with representative PRRSV-1 sequences available in GenBank (black). A) ORF2, B) ORF3, C) ORF4, D) ORF5, E) ORF6, F) ORF7. GenBank accession number is given, as well as country/identifier/year, if available. The trees are rooted with type 2 strain VR2332 (PRU87392). The percentage of trees in which the associated taxa clustered together is shown next to the branches (only >50).

Besides analysing the complete ORF2-ORF7 sequences of 74 Dutch PRRS viruses, the individual ORFs were also compared with sequences in GenBank in phylogenetic trees (Fig 3). For some viruses the phylogenetic topology varied per ORF. For instance, NL/GD-4-13/2015 was placed with viruses NL/GD-3-19/2014 and NL/GD-6-7/2016 in ORF5 and ORF6, whereas in ORF2, ORF3, ORF4 and ORF7 no clustering can be seen (Fig 3, highlighted in green). Another example is the clustering of NL/GD-3-11/2015 with NL/GD-2-9/2014 and NL/GD-3-7/2014 (Belgium-likes) in ORF2 and ORF3, whereas in ORF4, ORF5, ORF6 and ORF7 NL/GD-3-11/2015 seems to be a totally different virus (Fig 3, highlighted in purple). The most striking example is strain NL/GD-5-18/2015 that clustered with Austrian AUT/15-33/2015 (GenBank KU494019) in the phylogenetic trees constructed from complete ORF2, ORF3, ORF4 and ORF5 sequences (86.3–89.5% similarity), whereas in the ORF6 and ORF7 trees it clustered (96.2–96.9% identity) with Lelystad virus (Fig 3, highlighted in red; S2 Table).

## Discussion

In regional elimination and national eradication efforts PRRSV genotyping is one of the key tools to assess the performance of action to help improve internal and external biosecurity measures and to better understand the virus ecology. This manuscript describes the first extensive molecular study about Dutch PRRS viruses since its first description in the early nineties. The results demonstrate that all 74 PRRSV strains belong to PRRSV-1 (Fig 1), but the diversity among strains is high, based on the nucleotide identity (S1 appendix and S2 Table), individual ORF length (Fig 2 and S3 Table) and phylogenetic trees of individual ORFs (Fig 3). Furthermore, the most investigated viruses in this study form a distinct Dutch cluster based on ORF2-ORF7 sequences (Fig 1). The Netherlands is exporting approximately 10 million and importing half a million live pigs per year [37] and is endemic for PRRSV, like many other countries in Europe. Therefore, it is expected that with the animals, PRRS virus strains spread over these countries. Sampling bias is a probable clarification for this as many countries do not sequence and/or deposit their sequences in GenBank, resulting in incomplete databases for ORF2-ORF7. With an economic high impact disease like PRRS across country borders, monitoring of circulating viruses is an important tool that would greatly benefit from more frequent sharing of sequence data by different European countries, including the Netherlands.

The high genetic diversity of the presented viruses suggests that PRRSV is constantly evolving to adapt to naturally, as well as vaccine induced immunity as also previously described [38]. Despite vaccination of the majority of the pig population in the Netherlands, still many outbreaks are reported, and in literature it is already suggested to improve vaccines to overcome the disadvantages of current vaccines [39].

The nucleotide identity of individual ORFs with Lelystad virus (S1 appendix) confirms that ORF6 and ORF7 are the most conserved genes and justifies these targets in the diagnostic PRRS detection PCR [40]. However, a primer based approach is biased in advance. This was demonstrated by the fact that some of the viruses selected for ORF2-ORF7 sequencing based on strong positive results in the detection PCR, were not picked-up by the primers designed to obtain the ORF2-ORF7 amplicons. Even after designing two additional forward primers, not for all PRRSV isolates ORF2-ORF7 amplicons could be generated. Therefore, it is likely that the presented diversity of the viruses is an underestimation of the true diversity of PRRS viruses. As a result, as long as primer-based approaches are used for PRRS detection and sequencing, we must be aware that there will be viruses missed in routine testing.

Currently, PRRSV genotyping is being performed mostly based on ORF5 and/or ORF7 sequence analysis [41–44]. The data presented here shows that the phylogenetic topology of some viruses is ORF dependent, as also previously described [44]. For example, based on ORF5 alone, NL/GD-5-18/2015 would be categorized as a field virus (Fig 3D), whereas based on ORF6 or ORF7 the collected virus would be designated as a vaccine virus derivative (Fig 3E and 3F). A possible explanation for this ORF dependent clustering in phylogenetic trees is recombination [17]. PRRSV strains can recombine if coinfection of a cell with two or more strains occurs. So, the identity of a given PRRSV strain is largely incomplete if only ORF5 or ORF7 are known. Although whole genome sequencing of the PRRS viruses may give a more complete picture in support of the molecular epidemiology, sequencing ORF2-ORF7 is already an enormous improvement for PRRSV routine sequencing. Based on one ORF alone, the identity of the strain of interest might be misinterpreted and wrong conclusions may be drawn in a diagnostic and epidemiological perspective, as also recently observed [45].

Regarding PRRSV genome diversity studies, ORF5 is the most examined gene and one of the most variable regions of the genome [41, 44, 46]. Also in the present study, a high genetic diversity, when comparing the 74 Dutch sequences with Lelystad virus, was seen for ORF5 (S1 appendix and S2 Table). Although this was not due to its variation in length (Fig 2) or the amount of N-glycosylation sites (three putative sites for 69 of the 74 viruses; S4 Table).

Of the genome region that encodes for one of the structural proteins, ORF3 is the most variable part. The 3’-end of this region is prone for deletions as previously described [43, 44] and the deletions found in the present study always affected the open reading frame of ORF4. Not only the variation in nucleotide identity compared to the Lelystad strain (S1 appendix) was high, but the viruses studied varied also in their ORF3 length (Fig 2). In addition, another eight variants in length were observed at the other ORF3 (non-Dutch) sequences from GenBank used in Fig 1: 744, 750, 762, 765, 774, 780, 786 and 792 nucleotides, respectively. Although it is suggested that viruses with ORF3 deletions will be outcompeting nondeleted viruses in the field [43], our results show that besides shorter ORF3 genes, also longer ORF3 genes compared to Lelystad virus were observed (Fig 2). For ORF4, three variants in the Dutch strains (Fig 2) were observed, whereas the used GenBank sequences in Fig 1 contain another seven variants in length: 516, 519, 528, 534, 540, 546 and 555 nucleotides, respectively. The variation in length of ORF4 can be explained by the fact that the 5’-end of ORF4 and the 3’-end of ORF3 coding regions overlap and hence any deletion found in ORF3 will also affect ORF4 [47].

Future research will be focusing on PRRSV-1 recombination and its implication on genetic analysis. It is of upmost importance that the variation of circulating PRRSV is being monitored and genetically analyzed, in order to improve diagnostics, increase vaccine efficacy and identification of found virus strains to support internal and external biosecurity measures on local farms.

## Supporting information

S1 Appendix Genetic similarity (%) with Lelystad virus (LV). Comparison is based on ORF2-ORF7 nucleotide sequences and the individual ORFs sequences of 74 Dutch isolates collected in 2014–2016.
(TIF)

S1 Table PCR amplification primers and Sanger sequence primers. (XLSX)

S2 Table Genetic similarity (%) with Lelystad virus. (XLSX)

S3 Table Individual ORF length of each of the 74 Dutch viruses. (XLSX)

S4 Table Putative N-glycosylation sites and signal peptides of ORF2, ORF3, ORF4 and ORF5 of each of the 74 Dutch viruses. (XLSX)

## References

[1] Zimmerman JJ. Porcine reproductive and respiratory syndrome virus (porcine arterivirus). In: Zimmerman JJ, Karriker LA, Ramirez A, Schwartz KJ, Stevenson GW, editors. Diseases of Swine. 10 ed2010.

[2] Duinhof TF, van Schaik G, van Esch EJ, Wellenberg GJ. Detection of PRRSV circulation in herds without clinical signs of PRRS: comparison of five age groups to assess the preferred age group and sample size. Vet Microbiol. 2011;150(1–2):180–4. 10.1016/j.vetmic.2011.01.001 . 21273010

[3] Kvisgaard LK. Porcine Reproductive and Respiratory Syndrome Virus (PRRSV) [PhD]. Frederiksberg, Denmark: National Veterinary Institute, Technical University of Denmark; 2013.

[4] Rossow KD, Bautista EM, Goyal SM, Molitor TW, Murtaugh MP, Morrison RB, et al Experimental porcine reproductive and respiratory syndrome virus infection in one-, four-, and 10-week-old pigs. J Vet Diagn Invest. 1994;6(1):3–12. 10.1177/104063879400600102 . 8011777

[5] Terpstra C, Wensvoort G, Pol JM. Experimental reproduction of porcine epidemic abortion and respiratory syndrome (mystery swine disease) by infection with Lelystad virus: Koch's postulates fulfilled. Vet Q. 1991;13(3):131–6. 10.1080/01652176.1991.9694297 . 1949539

[6] Cavanagh D. Nidovirales: a new order comprising Coronaviridae and Arteriviridae. Arch Virol. 1997;142(3):629–33. . 9349308

[7] Conzelmann KK, Visser N, Van Woensel P, Thiel HJ. Molecular characterization of porcine reproductive and respiratory syndrome virus, a member of the arterivirus group. Virology. 1993;193(1):329–39. 10.1006/viro.1993.1129 . 8438574 PMC7131490

[8] Meng XJ, Paul PS, Halbur PG, Lum MA. Phylogenetic analyses of the putative M (ORF 6) and N (ORF 7) genes of porcine reproductive and respiratory syndrome virus (PRRSV): implication for the existence of two genotypes of PRRSV in the U.S.A. and Europe. Arch Virol. 1995;140(4):745–55. . 7794115 10.1007/BF01309962 PMC7086766

[9] Allende R, Lewis TL, Lu Z, Rock DL, Kutish GF, Ali A, et al North American and European porcine reproductive and respiratory syndrome viruses differ in non-structural protein coding regions. J Gen Virol. 1999;80 (Pt 2):307–15. 10.1099/0022-1317-80-2-307 . 10073689

[10] Fang Y, Schneider P, Zhang WP, Faaberg KS, Nelson EA, Rowland RR. Diversity and evolution of a newly emerged North American Type 1 porcine arterivirus: analysis of isolates collected between 1999 and 2004. Arch Virol. 2007;152(5):1009–17. 10.1007/s00705-007-0936-y . 17323198

[11] Forsberg R. Divergence time of porcine reproductive and respiratory syndrome virus subtypes. Mol Biol Evol. 2005;22(11):2131–4. 10.1093/molbev/msi208 . 16000650

[12] Nelsen CJ, Murtaugh MP, Faaberg KS. Porcine reproductive and respiratory syndrome virus comparison: divergent evolution on two continents. J Virol. 1999;73(1):270–80. 9847330 10.1128/jvi.73.1.270-280.1999 PMC103831

[13] Gao ZQ, Guo X, Yang HC. Genomic characterization of two Chinese isolates of porcine respiratory and reproductive syndrome virus. Arch Virol. 2004;149(7):1341–51. 10.1007/s00705-004-0292-0 . 15221535

[14] Nam E, Park CK, Kim SH, Joo YS, Yeo SG, Lee C. Complete genomic characterization of a European type 1 porcine reproductive and respiratory syndrome virus isolate in Korea. Arch Virol. 2009;154(4):629–38. 10.1007/s00705-009-0347-3 . 19296201

[15] Ropp SL, Wees CE, Fang Y, Nelson EA, Rossow KD, Bien M, et al Characterization of emerging European-like porcine reproductive and respiratory syndrome virus isolates in the United States. J Virol. 2004;78(7):3684–703. 10.1128/JVI.78.7.3684-3703.2004 15016889 PMC371078

[16] Stadejek T, Stankevicius A, Storgaard T, Oleksiewicz MB, Belak S, Drew TW, et al Identification of radically different variants of porcine reproductive and respiratory syndrome virus in Eastern Europe: towards a common ancestor for European and American viruses. J Gen Virol. 2002;83(Pt 8):1861–73. 10.1099/0022-1317-83-8-1861 . 12124450

[17] Kappes MA, Faaberg KS. PRRSV structure, replication and recombination: Origin of phenotype and genotype diversity. Virology. 2015;479–480:475–86. 10.1016/j.virol.2015.02.012 . 25759097 PMC7111637

[18] Wu WH, Fang Y, Farwell R, Steffen-Bien M, Rowland RR, Christopher-Hennings J, et al A 10-kDa structural protein of porcine reproductive and respiratory syndrome virus encoded by ORF2b. Virology. 2001;287(1):183–91. 10.1006/viro.2001.1034 . 11504553

[19] Johnson CR, Griggs TF, Gnanandarajah J, Murtaugh MP. Novel structural protein in porcine reproductive and respiratory syndrome virus encoded by an alternative ORF5 present in all arteriviruses. J Gen Virol. 2011;92(Pt 5):1107–16. 10.1099/vir.0.030213-0 21307222 PMC3139420

[20] Kapur V, Elam MR, Pawlovich TM, Murtaugh MP. Genetic variation in porcine reproductive and respiratory syndrome virus isolates in the midwestern United States. J Gen Virol. 1996;77 (Pt 6):1271–6. 10.1099/0022-1317-77-6-1271 . 8683216

[21] Stadejek T, Oleksiewicz MB, Potapchuk D, Podgorska K. Porcine reproductive and respiratory syndrome virus strains of exceptional diversity in eastern Europe support the definition of new genetic subtypes. J Gen Virol. 2006;87(Pt 7):1835–41. 10.1099/vir.0.81782-0 . 16760385

[22] Stadejek T, Oleksiewicz MB, Scherbakov AV, Timina AM, Krabbe JS, Chabros K, et al Definition of subtypes in the European genotype of porcine reproductive and respiratory syndrome virus: nucleocapsid characteristics and geographical distribution in Europe. Arch Virol. 2008;153(8):1479–88. 10.1007/s00705-008-0146-2 . 18592131

[23] Wensvoort G, Terpstra C, Pol JM, ter Laak EA, Bloemraad M, de Kluyver EP, et al Mystery swine disease in The Netherlands: the isolation of Lelystad virus. Vet Q. 1991;13(3):121–30. 10.1080/01652176.1991.9694296 . 1835211

[24] Cay A, Wellenberg GJ, Kerkhofs P, editors. Phylogenetic analysis of Dutch and Belgian EU-type PRRSV strains. International Pig Veterinary Society Congress (IPVS); 2008 6 22–26, 2008; Durban, South Africa.

[25] Wellenberg GJ, Cruijsen T, editors. Phylogenetic analysis of Dutch PRRSV strains. International pig veterinary society congress (IPVS); 2010 7 18–21, 2010; Vancouver, Canada.

[26] Donadeu M, Arias M, Gomez-Tejedor C, Aguero M, Romero LJ, Christianson WT, et al Using polymerase chain reaction to obtain PRRSV-free piglets from endemically infected herds. Swine Health and Production. 1999;7(6):7.

[27] Buter GJ, Dortmans JCFM. Magmax pathogen RNA: serum protocol: protocols.io; 2019 [cited 2019 18-4-2019]. Available from: 10.17504/protocols.io.z55f886

[28] Nielsen HS, Liu G, Nielsen J, Oleksiewicz MB, Botner A, Storgaard T, et al Generation of an infectious clone of VR-2332, a highly virulent North American-type isolate of porcine reproductive and respiratory syndrome virus. J Virol. 2003;77(6):3702–11. 10.1128/JVI.77.6.3702-3711.2003 12610145 PMC149535

[29] Buter GJ, Dortmans JCFM. Protocol cDNA synthesis of PRRSV: Protocols.io; 2019 [cited 2019 18-4-2019]. Available from: 10.17504/protocols.io.z54f88w

[30] Buter GJ, Dortmans JCFM. Amplification PCR of ORF2-ORF7 of PRRSV: protocols.io; 2019 [cited 2019 18-4-2019]. Available from: 10.17504/protocols.io.z53f88n

[31] Tamura K, Stecher G, Peterson D, Filipski A, Kumar S. MEGA6: Molecular Evolutionary Genetics Analysis version 6.0. Mol Biol Evol. 2013;30(12):2725–9. 10.1093/molbev/mst197 24132122 PMC3840312

[32] Sievers F, Wilm A, Dineen D, Gibson TJ, Karplus K, Li W, et al Fast, scalable generation of high-quality protein multiple sequence alignments using Clustal Omega. Mol Syst Biol. 2011;7:539 10.1038/msb.2011.75 21988835 PMC3261699

[33] Vigerust DJ, Shepherd VL. Virus glycosylation: role in virulence and immune interactions. Trends Microbiol. 2007;15(5):211–8. 10.1016/j.tim.2007.03.003 . 17398101 PMC7127133

[34] Dokland T. The structural biology of PRRSV. Virus Res. 2010;154(1–2):86–97. 10.1016/j.virusres.2010.07.029 . 20692304 PMC7114433

[35] Gupta R, Brunak S. Prediction of glycosylation across the human proteome and the correlation to protein function. Pac Symp Biocomput. 2002:310–22. . 11928486

[36] Petersen TN, Brunak S, von Heijne G, Nielsen H. SignalP 4.0: discriminating signal peptides from transmembrane regions. Nat Methods. 2011;8(10):785–6. 10.1038/nmeth.1701 . 21959131

[37] Government D. Import- en exportcijfers varkens per land: CKAN; 2019 [updated 21-12-2018; cited 2019 14-3-2019]. Available from: https://www.rvo.nl/sites/default/files/2018/04/Import-export-varkens.xls.

[38] Morgan SB, Graham SP, Salguero FJ, Sanchez Cordon PJ, Mokhtar H, Rebel JM, et al Increased pathogenicity of European porcine reproductive and respiratory syndrome virus is associated with enhanced adaptive responses and viral clearance. Vet Microbiol. 2013;163(1–2):13–22. 10.1016/j.vetmic.2012.11.024 . 23313323

[39] Nan Y, Wu C, Gu G, Sun W, Zhang YJ, Zhou EM. Improved Vaccine against PRRSV: Current Progress and Future Perspective. Front Microbiol. 2017;8:1635 10.3389/fmicb.2017.01635 28894443 PMC5581347

[40] Wernike K, Bonilauri P, Dauber M, Errington J, LeBlanc N, Revilla-Fernandez S, et al Porcine reproductive and respiratory syndrome virus: interlaboratory ring trial to evaluate real-time reverse transcription polymerase chain reaction detection methods. J Vet Diagn Invest. 2012;24(5):855–66. 10.1177/1040638712452724 . 22807507

[41] Shi M, Lam TT, Hon CC, Hui RK, Faaberg KS, Wennblom T, et al Molecular epidemiology of PRRSV: a phylogenetic perspective. Virus Res. 2010;154(1–2):7–17. 10.1016/j.virusres.2010.08.014 . 20837072

[42] Murtaugh MP, Stadejek T, Abrahante JE, Lam TT, Leung FC. The ever-expanding diversity of porcine reproductive and respiratory syndrome virus. Virus Res. 2010;154(1–2):18–30. 10.1016/j.virusres.2010.08.015 . 20801173

[43] Oleksiewicz MB, Botner A, Toft P, Grubbe T, Nielsen J, Kamstrup S, et al Emergence of porcine reproductive and respiratory syndrome virus deletion mutants: correlation with the porcine antibody response to a hypervariable site in the ORF 3 structural glycoprotein. Virology. 2000;267(2):135–40. 10.1006/viro.1999.0103 . 10662609

[44] Darwich L, Gimeno M, Sibila M, Diaz I, de la Torre E, Dotti S, et al Genetic and immunobiological diversities of porcine reproductive and respiratory syndrome genotype I strains. Vet Microbiol. 2011;150(1–2):49–62. 10.1016/j.vetmic.2011.01.008 . 21310555

[45] Franzo G, Cecchinato M, Martini M, Ceglie L, Gigli A, Drigo M. Observation of high recombination occurrence of Porcine Reproductive and Respiratory Syndrome Virus in field condition. Virus Res. 2014;194:159–66. 10.1016/j.virusres.2014.08.005 . 25150757 PMC7127771

[46] Balka G, Podgorska K, Brar MS, Balint A, Cadar D, Celer V, et al Genetic diversity of PRRSV 1 in Central Eastern Europe in 1994–2014: origin and evolution of the virus in the region. Sci Rep. 2018;8(1):7811 10.1038/s41598-018-26036-w 29773820 PMC5958080

[47] Dea S, Gagnon CA, Mardassi H, Pirzadeh B, Rogan D. Current knowledge on the structural proteins of porcine reproductive and respiratory syndrome (PRRS) virus: comparison of the North American and European isolates. Arch Virol. 2000;145(4):659–88. . 10893147 10.1007/s007050050662 PMC7087215

