# Supplementary figures and images for: Molecular characterization of type 1 porcine reproductive and respiratory syndrome viruses (PRRSV) isolated in the Netherlands from 2014 to 2016
Source: PLoS One. 2019 Jun 27;14(6):e0218481. doi: 10.1371/journal.pone.0218481 (PMC6597066; doi:10.1371/journal.pone.0218481)

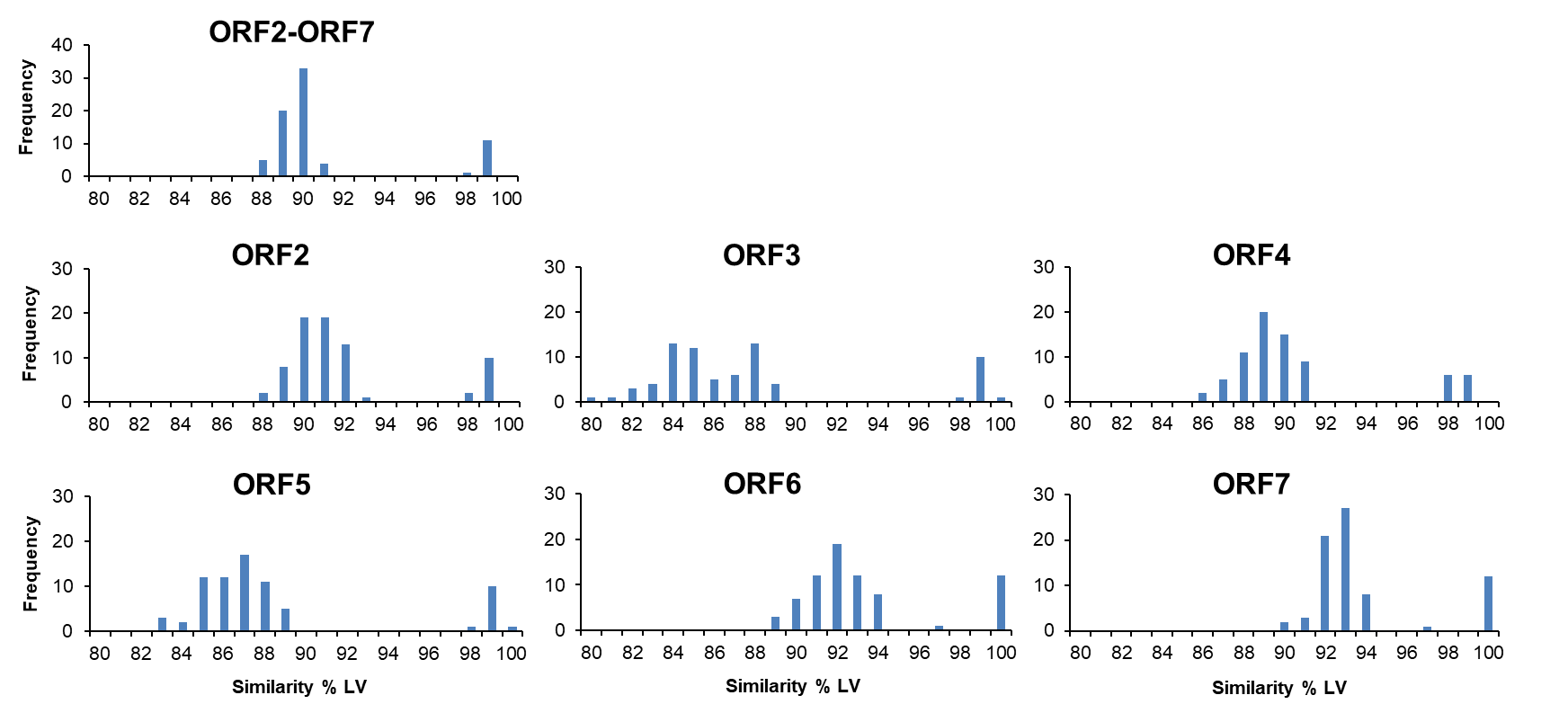

Supplement: S1 Appendix — Comparison is based on ORF2-ORF7 nucleotide sequences and the individual ORFs sequences of 74 Dutch isolates collected in 2014–2016. (TIF) [file pone.0218481.s001.tif]
